# Deoxynivalenol in the Gastrointestinal Tract of Immature Gilts under *per os* Toxin Application

**DOI:** 10.3390/toxins6030973

**Published:** 2014-03-05

**Authors:** Agnieszka Waśkiewicz, Monika Beszterda, Marian Kostecki, Łukasz Zielonka, Piotr Goliński, Maciej Gajęcki

**Affiliations:** 1 Department of Chemistry, Poznań University of Life Sciences, Wojska Polskiego 75, Poznań 60-625, Poland; E-Mails: agat@up.poznan.pl (A.W.); monika.beszterda@up.poznan.pl (M.B.); marian.kostecki@up.poznan.pl (M.K.); 2 Department of Veterinary Prevention and Feed Hygiene, University of Warmia and Mazury in Olsztyn, Olsztyn 10-719, Poland; E-Mails: lukasz.zielonka@uwm.edu.pl (Ł.Z.); gajecki@uwm.edu.pl (M.G.)

**Keywords:** deoxynivalenol, gastrointestinal tract, immature gilts, low doses, mycotoxicosis

## Abstract

Deoxynivalenol is also known as vomitoxin due to its impact on livestock through interference with animal growth and acceptance of feed. At the molecular level, deoxynivalenol disrupts normal cell function by inhibiting protein synthesis via binding to the ribosome and by activating critical cellular kinases involved in signal transduction related to proliferation, differentiation and apoptosis. Because of concerns related to deoxynivalenol, the United States FDA has instituted advisory levels of 5 µg/g for grain products for most animal feeds and 10 µg/g for grain products for cattle feed. The aim of the study was to determine the effect of low doses of deoxynivalenol applied *per os* on the presence of this mycotoxin in selected tissues of the alimentary canal of gilts. The study was performed on 39 animals divided into two groups (control, C; *n* = 21 and experimental, E; *n* = 18), of 20 kg body weight at the beginning of the experiment. Gilts received the toxin in doses of 12 µg/kg b.w./day (experimental group) or placebo (control group) over a period of 42 days. Three animals from two experimental groups were sacrificed on days 1, 7, 14, 21, 28, 35 and 42, excluding day 1 when only three control group animals were scarified. Tissues samples were prepared for high performance liquid chromatography (HPLC) analyses with the application of solid phase extraction (SPE). The results show that deoxynivalenol doses used in our study, even when applied for a short period, resulted in its presence in gastrointestinal tissues. The highest concentrations of deoxynivalenol reported in small intestine samples ranged from 7.2 (in the duodenum) to 18.6 ng/g (in the ileum) and in large intestine samples from 1.8 (in transverse the colon) to 23.0 ng/g (in the caecum). In liver tissues, the deoxynivalenol contents ranged from 6.7 to 8.8 ng/g.

## 1. Introduction

Toxigenic *Fusarium* species are common pathogens of wheat, triticale and other cereals worldwide [1,2,3]. Many of them attack a range of plant parts and stages, such as seedlings, heads, roots, stems and ears, resulting in severe reductions of grain yield, often ranging from 10% to 40% [4,5]. Consequently, when cereal plants are infected with fungi, there is a risk of grain contamination with secondary metabolites, *i.e.*, mycotoxins, and their subsequent transfer to feed and food [6,7]. A major problem associated with animal feed contaminated with mycotoxins is not acute disease, but rather the ingestion of low levels of toxins, which may cause an array of metabolic, physiologic and immunologic disturbances [8,9,10]. Major *Fusarium* toxins found under the climatic conditions of Poland are deoxynivalenol, zearalenone, moniliformin and fumonisins detected in cereals during plant growth and vegetation [5,6,11,12]. The presence of deoxynivalenol (DON) in agricultural crops is an increasingly common problem associated with the occurrence of *Fusarium* head blight (FHB) infection not only under temperate weather conditions, possibly because of the extensive application of “no-tillage farming”, changing climate patterns and simultaneously, enhanced cultivation of host crops such as maize and wheat [13]. Numerous studies indicated that choosing a less susceptible cultivar is a powerful tool to ensure a low DON concentration in cereal grain even under highly infectious conditions. This strategy enables farmers to make use of the benefits of conservation tillage and, simultaneously, produce high quality grain [14].

Structurally, DON is a polar organic compound with the IUPAC name: 12,13-epoxy-3α,7α,15-trihydroxytrichothec-9-en-8-on [15,16]. The ketone position in C_8_ is a characteristic of the class B trichothecenes, also the number and position of the hydroxyl and acetyl-ester groups can influence the relative toxicity within cells. In addition, via the epoxy group, this toxin is able to bind to the large subunit of eukaryotic ribosomes and interfere with peptidyl transferase, thus impairing initiation or elongation of peptide chains [17,18]. DON relative capacity to interfere with protein synthesis has been attributed to a combination of different factors: the rate of transport into cells, metabolism by cytosol enzymes, changes in affinity to the active binding site or capacity to interfere with protein synthesis. 

The intensity of DON toxic effects depends on the dose, species, duration of consumption, toxin purity and the route of administration [19,20]. Data on genotoxic effects of trichothecenes are scarce and these toxins are classified to group 3 (inadequate evidence) by the International Agency on Cancer Research. Among various animal species, swine are known to be especially susceptible to DON and could therefore serve as a model for human sensitivity to this mycotoxin. DON absorption in pigs is rapid, reaching peak plasma concentrations within 15 to 30 min of dosing [18,21]. Up to 0.82 systemic absorption was recorded in pigs administered DON orally. Transient tissue distribution of DON in pigs occurs with an elimination half-life of 3.9 h and very limited accumulation in tissues. From numerous studies on laboratory animals and cell lines it was concluded that low dose exposure upregulates expression of cytokines, chemokines and inflammatory genes with a concurrent immune stimulation. In addition, low to moderate dose acute oral exposure to trichothecenes causes vomiting, diarrhea, gastroenteritis, anorexia, reduced weight gain, malabsorption of glucose, glutamine and 5-methyltetrahydrofolic acid, oesophageal perforation, circulatory shock and can ultimately lead to death [22,23,24]. DON affects the integrity of intestinal epithelium through alterations in cell morphology and differentiation in the barrier function. In turn, high dose exposure promotes leukocyte apoptosis with a concomitant immune suppression and severe damage to the lymphoid and epithelial cells of the gastrointestinal mucosa resulting in hemorrhage, endotoxemia and shock [17,25,26,27]. Other targets include bone marrow and thymus, which can contribute to generalized immunosuppression. Interestingly, these gut effects can occur in animals exposed to trichothecenes via inhalation. The United States Food and Drug Administration (USFDA) has established Advisory Levels for DON in grain and grain by-products based on the intended use [18]. For beef, feedlot cattle older than 4 months and chickens it is 10 mg/kg while for pigs and all other animal species it is 5 mg of DON per kg of fodder.

The gastrointestinal tract is the first barrier against feed contaminants as well as the first target for mycotoxins. The intestine is a preferential immune site where immunoregulatory mechanisms simultaneously defend the body against pathogens, but also maintain tissue homeostasis to avoid immune-mediated pathology in response to environmental challenges [28]. However, the toxin has been demonstrated to readily cross the epithelial barrier by a paracellular pathway [29]. Hence, the purpose of the present study was to examine the effect of *per os* DON exposure at NOAEL (no observed adverse effect level) doses on the absorption, accumulation and final presence of this toxin in tissues of the gastrointestinal tract of gilts, *i.e.*, the liver, the small and large intestine, using high performance liquid chromatography tools. 

## 2. Results and Discussion

In the last two decades, many studies have described DON toxicity using diverse species as models, where DON consumption was shown to affect numerous physiological functions such as food intake, reproduction or immunity [30,31,32]. The presented experiment involved two groups of immature gilts (with body weight of up to 20 kg), which were orally administered deoxynivalenol at 12 μg/kg b.w. (group E, *n* = 18) or placebo (group C, *n* = 21) over a period of 42 days. The concentration of the toxin in the small intestine, large intestine and liver tissues was chromatographically analyzed. Until now, most performance and toxicological data in pigs have been obtained with medium to high doses of the toxin, *i.e.*, 2 to 10 mg/kg of feed [33,34]. A survey of 11,022 cereal samples from 12 European countries showed that 57% were positive for DON, with 7% containing DON concentrations of 750 µg/kg or greater. One of the most important physicochemical properties of DON is its ability to withstand high temperatures, which poses a risk of its occurrence in feed and food [35]. *Ipso facto*, it is known that feed and food processing may have no effect on DON concentrations in the final product. Additionally, it seems possible that differences in the feed form could modulate the bioavailability of DON, as it could affect the liberation of the toxin from the matrix and thereby influence residue concentration in the animal tissue. In this study, the daily DON intake during the whole period of the experiment reflects slight differences between animals because of DON doses converted into body weight and precisely administered by a small portion of the feed matrix.

### 2.1. DON Residues in Small Intestine Tissues

The effect of feed contamination with low doses of DON on the presence of the toxin was investigated in tissue preparations from the duodenum, jejunum and ileum. As described in Table 1, in this experiment, diets significantly affect tissue concentrations of the toxin. Under individual intoxication of DON with 12 µg/kg b.w. per day doses, the toxin content in small intestine tissues ranged from 0.00 (terms I and II) to 18.60 ng/g (term VI) (Table 1). At terms I and II no mycotoxins were detected in any samples analyzed. The highest DON concentrations were detected during terms of slaughter IV–VI, at 7.20 ng/g for the duodenum, 9.20 ng/g for the jejunum and 18.60 ng/g for the ileum samples, respectively. Only in the ileum samples the content of DON in diets for pigs demonstrated linear dose relationships to tissue concentration. However, these relationships were characterized by high inter-individual variation.

**Table 1:** Concentration levels of deoxynivalenol (DON) [ng/g] with determined homogenous groups (*α =* 0.05) and the derived carry over factor of DON in the first section of gastrointestinal tract-small intestine of gilts fed diets containing NOAEL (12 µg/kg b.w./day) concentrations of toxin.

Days of the experiment	Total doses [µg/kg b.w.]	DON amounts in small intestine [ng/g] ± standard deviation					
		Duodenum	Carry over factor	Jejunum	Carry over factor	Ileum	Carry over factor
7 (term I)	84	0 ^a^ ± 0	0	0 ^a^ ± 0	0	0 ^a^ ± 0	0
14 (term II)	168	0 ^a^ ± 0	0	0 ^a^ ± 0	0	0 ^a^ ± 0	0
21 (term III)	252	6.71 ^b^ ± 0.22	0.027	1.80 ^a,b^ ± 0.64	0.007	0 ^a^ ± 0	0
28 (term IV)	336	7.20 ^b^ ± 0.16	0.021	9.20 ^c^ ± 2.54	0.027	9.20 ^b^ ± 4.65	0.027
35 (term V)	420	4.24 ^b^ ± 1.57	0.010	4.54 ^b^ ± 0.95	0.011	11.19 ^b,c^ ± 2.40	0.027
42 (term VI)	504	6.13 ^b^ ± 2.13	0.012	8.06 ^c^ ± 1.08	0.016	18.60 ^c^ ± 4.26	0.037

Note: The same symbols for average values in a particular column indicate no statistically significant differences in the level of DON concentrations within the analyzed samples (Tukey HSD test).

Dänicke and co-workers [36] indicated that only about 1% of ingested DON reaches the proximal parts of the small intestine (duodenum and jejunum) in pigs with maximum DON contents of 1.3 mg/kg freeze-dried ingesta when swine weighing 88 kg were fed 1.1 kg feed with a DON concentration of 4.2 mg/kg. However, also lower and fluctuating DON accumulations can lead to morphological modifications of the intestinal epithelium after DON exposure *in vivo* [37]. In an experiment by Goyarts and co-workers [38], 15 min after *per os* exposure DON was found in all serum samples. This early detection of DON in serum [39] showed that the absorption of DON was rapid and indicated that it could start in the stomach or in the upper part of duodenum [40]. Presented arguments may explain the observed gradual saturation of tissues, which eventually led to an increased DON concentration in successive sections of the alimentary tract in proportion to the passing time. In turn, we would also have to remember that these slight DON levels, while gradually saturating tissues did not cause any increase in DON concentrations in earlier sections of the alimentary tract with the passage of time. Such a situation could be attributed to the acquisition of food tolerance, consisting in the inhibition of inflammation processes in the alimentary tract, particularly the ileum and the descending colon [41]. This might be a certain “escape” mechanism of mycotoxins before the induction of the local immune system, similarly as it is the case with Tregs induction by classical pathogens [42] during chronic infections.

Using the obtained results, the carry-over factor was calculated as the quotient of toxin concentration in the tissue (µg/kg) by toxin concentration in the diet (µg/kg), included as the total administered doses in the defined term experiment, according to previous researchers [38,43,44]

The mean carry-over factors in the first and second terms amounted to 0, while the maximum values in that period of time were observed in samples from terms III (for the duodenum–0.027), IV (for the jejunum–0.027) and VI (for the ileum–0.037) (Table 1). For the duodenum and jejunum specimens the median carry-over factors decrease with increasing proportions of exposure to DON starting from term IV for the duodenum and term V for the jejunum. The summary carry-over factor for the duodenum is higher than for the jejunum and ileum, respectively. In a study by Goyarts and co-workers [38] the mean carry-over factor of DON and de-epoxy-DON, presented as the content of both substances in the specimen divided by the toxin content in the diet, for all swine decreased in the descending order from the bile (0.1046), kidneys (0.0151), liver (0.0057), serum (0.0023), muscle (0.0016) to back fat (0.0002). 

**Figure 1:**
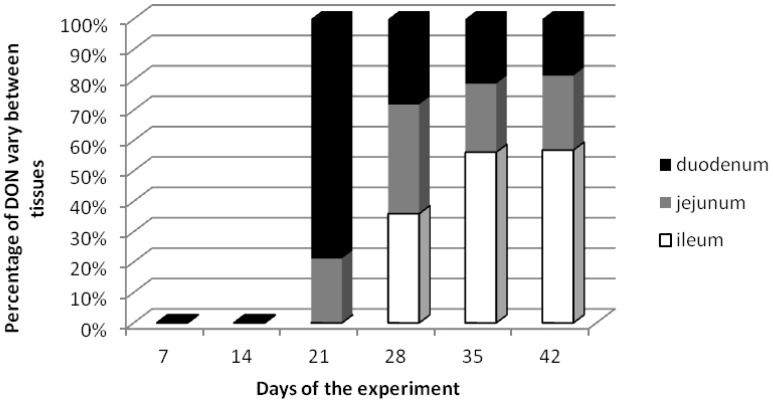
The percentage distribution of DON in tissues of small intestine (duodenum, jejunum, ileum).

The percentage distribution of DON in analyzed small intestine tissues is shown in Figure 1. Starting from date 3 the quantitative participation of the mycotoxin in different tissues changed. At term III the highest DON concentration was detected in the duodenum, while at term IV the same percentage of DON was recorded in the jejunum and ileum. At terms V and VI the highest DON contents were detected in the ileum samples at a simultaneous similar participation of DON in the duodenum and jejunum specimens. 

### 2.2. DON Residue in Large Intestine and Liver Tissues

Using individual intoxication of DON the toxin was not detected in transverse and descending colon tissues collected in the first three dates of the experiment (Table 2). Up to term IV of sample collection the highest DON amounts were observed in the liver tissues (7.90 ng/g), and later in the ascending and descending colon sections (terms V and VI). The concentration of DON for different fragments of the gastrointestinal tract fell within the following ranges (in ng/g): for the liver from 6.70 (term VI) to 8.80 (term V), for the cecum from 0.0 (term I) to 20.5 (term V), for the transverse colon from 0.0 (terms I, II, III, V and VI) to 1.8 (term IV), and for the descending colon from 0.0 (terms I, II and III) to 20.00 (term VI). Averaging the recorded DON concentrations in tissues from all study terms the highest average level of the toxin was 8.70 ng/g in the cecum samples. 

**Table 2:** DON concentration [ng/g] with determined homogenous groups (*α* = 0.05) and the resulting carry-over factor of DON in the second section of the gastrointestinal tract, *i.e.*, the large intestine, of gilts fed diets containing NOAEL concentrations of DON.

Days of the experiment	Total doses [µg/kg b.w.]	DON amounts in large intestine [ng/g] ± standard deviation							
		Cecum	Carry over factor	Ascending colon	Carry over factor	Transverse colon	Carry over factor	Descending colon	Carry over factor
7 (term I)	84	0 ^a^ ± 0	0	0 ^a^ ± 0	0	0 ^a^ ± 0	0	0 ^a^ ± 0	0
14 (term II)	168	3.70 ^a,b^ ± 0.47	0.022	2.50 ^a^ ± 0.17	0.015	0 ^a^ ± 0	0	0 ^a^ ± 0	0
21 (term III)	252	6.42 ^a,b^ ± 0.22	0.025	3.10 ^a^ ± 1.56	0.012	0 ^a^ ± 0	0	0 ^a^ ± 0	0
28 (term IV)	336	9.81 ^b^ ± 4.08	0.029	7.44 ^a^ ± 0.92	0.022	1.80 ^b^ ± 0.18	0.005	6.75 ^b^ ± 2.53	0.020
35 (term V)	420	9.29 ^b^ ± 2.54	0.022	20.52 ^b^ ± 5.77	0.049	0 ^a^ ± 0	0	10.92 ^b^ ± 2.62	0.026
42 (term VI)	504	23.00 ^c^ ± 4.89	0.046	16.09 ^b^ ± 3.57	0.032	0 ^a^ ± 0	0	20.00 ^c^ ± 3.27	0.040

Note: The same symbols for average values in a particular column indicate no statistically significant differences in the level of DON concentrations within the analyzed samples (Tukey HSD test).

In a similar study the concentrations of DON decreased from bile > kidney > serum > liver = muscle in an experiment, where pigs received diets containing 0%, 25% and 50% of contaminated wheat containing 2.5 mg DON/kg [43].

The median carry-over factors for the large intestine samples showed significant differences. The maximum stability for the observed indicator was found for the cecum samples. The higher values of the carry-over factor were recorded in term VI for the cecum (0.046), term V for the ascending colon (0.049), term IV for the transverse colon and term VI for the descending colon (0.040) (Table 2).

The summary carry-over factor for the cecum was higher than for the ascending, descending and transverse colon, respectively. For the liver the median carry-over factors ranged from 0.013 to 0.081. The decreasing factor values were only observed in these tissue samples during the experimental periods (Table 3).

**Figure 2:**
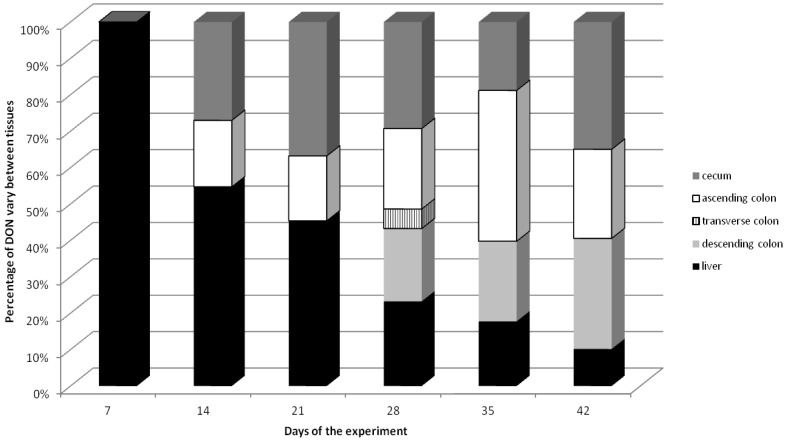
The percentage distribution of DON in large intestine (cecum, ascending colon, transverse colon and descending colon) and liver samples.

The percentage distribution of DON in the large intestine and liver tissue samples is shown in Figure 2. From term II to term III the highest percentages of the toxin with a downward trend were observed in the liver tissues, whereby starting from term IV the amounts of DON in the analyzed samples indicate similar levels in all tissues.

**Table 3:** Concentration levels of DON [ng/g] with determined homogenous groups (*α =* 0.05) and the derived carry over factor of DON in liver of gilts fed diets containing NOAEL concentrations of DON.

Days of the experiment	Total doses [µg/kg b.w.]	DON amounts in liver [ng/g] ± standard deviation	
		Liver	Carry over factor
7 (term I)	84	6.79 ^a^ ± 1.28	0.081
14 (term II)	168	7.51 ^a^ ± 0.74	0.045
21 (term III)	252	7.90 ^a^ ± 2.87	0.031
28 (term IV)	336	7.78 ^a^ ± 3.35	0.023
35 (term V)	420	8.80 ^a^ ± 3.69	0.021
42 (term VI)	504	6.70 ^a^ ± 4.28	0.013

Note: The same symbols for average values in a particular column indicate no statistically significant differences in the level of DON concentrations within the analyzed samples (Tukey HSD test).

The observed situation is confirmed by the results of the authors’ investigations [41], showing that the action of DON is multifaceted, e.g., it is an inhibitory factor for mRNA expression processes of the gene controlling the NOS-1 constitutive isomer and the NOS-2 inducible isomer. This causes specific changes in the functioning of the alimentary tract in gilts due to a reduced amount of NO, which is an inhibitor of neurotransmitters of the non-adrenergic and non-cholinergic systems [45,46]. As a consequence, low NO levels cause accelerated peristalsis of the oesophagus, stomach and intestines, inhibit stomach accommodation processes (relaxation, absorption, adaptation and successive contraction) and increased tension of intestinal sphincters, thus contributing to inhibition of gastric emptying and transfer of digesta in the intestines [47], *i.e.*, proliferation processes are activated [48]. All the presented situations are factors promoting enhanced biotransformation depending on energy resources of the organism [49], as well as better explain the cytotoxic properties of DON in the colon [50], which cytotoxicity in small doses is lower [51].

Up to now, exposure of swine to low doses of deoxynivalenol was investigated in the context of hematological, biochemical, immunological and histopathological parameters [9,52,53,54]. The toxicological principle of deoxynivalenol action may be similar in several organs, such as the lungs, liver or kidneys, and the toxin may cause damage and increase cell death in porcine intestinal cells, which is associated with metabolic stress [53]. Disturbed intestinal functions after DON ingestion seem to be connected with the fact that intestinal epithelial cells can be exposed to high luminal DON contents for a prolonged time following ingestion of contaminated feed [52,55]. An experiment on the cytotoxic effect of DON on a porcine intestinal cell line (IPEC-J2) demonstrated that the minimal effective dose to induce the cytotoxic effect was between 0.5 to 2.5 µM of DON solution in ethanol, whereas at a concentration of 10 µM this toxin caused cell damage, including rounding of cells, autolysis and cell loss from the monolayer. Such observations are confirmed by the currently unpublished results of a our study, from which it results that these low DON doses are only slightly cytotoxic to the local bacterial flora. It may be assumed that in the last segment of the large intestine (descending colon) the cytotoxic activity of DON is increased, but only at low doses [51] and the activity of fecal enzymes increases. In other words, a situation may occur promoting the occurrence of pathological changes in the final section of the porcine alimentary tract [56]. In addition, a significant decrease in ATP levels was observed at 48 h in a dose-dependent manner. Experimental acute DON toxicity in pigs includes digestive symptoms with emesis starting after 2 h following intravenous injection, together with increased defecation and diarrhea [57]. DON at high doses exceeding causes jejunal lesions, diagnosed after a 4-h exposure in an *ex-vivo* pig culture model including shortened and coalesced villi, lysis of enterocytes, and edema [58]. Similarly, at 4 mg/kg diet DON may cause corrugation in the fundic region of the stomach and after 5-week exposure to DON at 2.8 mg/kg diet, in the absence of body weight modulation in pigs, significant histopathological changes were observed [59]. Additionally, multifocal atrophy and villi fusion, apical necrosis of villi, cytoplasmatic vacuolation of enterocytes and edema of the lamina propria were observed in the jejunum and ileum of pigs administered DON [60]. In another study, Pinton and co-workers showed that DON causes a dose-dependent translocation of a pathogenic strain of *Escherichia coli* across the porcine IPEC-1 epithelial cell monolayers [61]. Similarly, an increased translocation of *Salmonella Thyphimurium* was observed in porcine IPEC-J2 exposed to low doses of DON [62].

Our experiments in combination with the results discussed above provide a significant contribution to the knowledge on DON toxicity and distribution in individual tissues of the porcine alimentary tract. This is the first report concerning quantitative analysis of deoxynivalenol in the gastrointestinal tract of gilts after *per os* toxin application and may be an important addition to knowledge concerning the effect of *per os* exposure to this toxin.

Summing up the presented results as well as studies conducted by other authors certain conclusions may be drawn. Accumulation of low DON exposure doses in the final section of the alimentary tract may contribute to specific situations, not necessarily pathological [48]. One of the may be connected with the cytotoxic property of DON. As a consequence, the count of microbial pathogens decreases, which constitute one of the proinflammatory factors in the large intestine [63]. On the other hand, exposure of animal organisms to low doses of the mycotoxin is a factor inhibiting mRNA expression of NOS controlling genes [41,46]. In this situation, we might propose a conclusion that low DON doses (below NOAEL) may have a therapeutic effect [51].

## 3. Experimental Section

All of the experimental procedures involving animals were carried out in compliance with the Polish legal regulations determining the terms and methods for performing experiments on animals (opinion of the local Ethics Committee for Animal Experimentation No. 88/2009).

### 3.1. Experimental Animals

This part of the study was conducted at the Department of Veterinary Prevention and Feed Hygiene, Faculty of Veterinary Medicine, the University of Warmia and Mazury in Olsztyn, Poland, using 39 clinically healthy female gilts. The mean initial body weight was ±20 kg. The gilts were housed in individual cages with an *ad libitum* access to water. The experimental diets were prepared locally and formulated according to the energy and amino acid requirements for piglets. The feed used in the present study was contaminated only with DON, which was confirmed by chromatography analysis of the feed matrix.

### 3.2. Experimental Design

A total of 39 animals were divided into two groups: control (*n* = 21) and experimental (*n* = 18). The animals from the experimental group were administered DON daily at a dose of 12 µg/kg body weight *per os* for 42 days. Analytical samples of the mycotoxin were applied in gelatin capsules before morning feeding. The animals from the control group were orally administered placebo (gelatin capsules without mycotoxin) for the same period of time as the experimental group. Amounts of toxin application were dependent on the body weight and updated weekly. Three animals from both groups (experimental and control) were sacrificed on days 1, 7, 14, 21, 28, 35 and 42 (a total of 6 gilts on each day), excluding day 1 when only three control group animals were sacrificed. Every week tissue samples were collected from the porcine gastrointestinal tract (the liver–left lobe, duodenum–first and middle sections, jejunum–middle section, ascending colon–middle section, descending colon–middle section).

### 3.3. Chemicals

The deoxynivalenol standard and organic solvents (HPLC grade) were purchased with a standard grade certificate from Sigma-Aldrich (Steinheim, Germany). All chemicals used for extraction and purification of DON were purchased from POCh (Gliwice, Poland). Water for the HPLC mobile phase was purified using a Milli-Q system (Millipore, Bedford, MA, USA).

### 3.4. Tissue Samples

Post-mortem samples of the following tissues: the duodenum, jejunum, ileum, cecum, ascending colon, transverse colon, descending colon and the liver were collected after rinsing with phosphate buffer. Pure tissues were stored at −80 °C until the shredding and extraction procedure.

### 3.5. Extraction and Purification Procedure

Tissue fragments (2 g) were homogenized twice with methanol, then centrifuged (4000 rpm) and evaporated. The residue was diluted in 20 mL water and 20 mL 0.2 M CH_3_COONa and applied on the top of immunoaffinity columns according to the manufacturer’s recommendations (IAC, VICAM, Watertown, MA, USA). The toxin was eluted with a mixture of acetonitrile: water (82:12, v/v). The eluate was evaporated to dryness at 40 °C under a stream of nitrogen. Dry residue was stored at −20 °C until HPLC analyses.

### 3.6. HPLC Analysis of Deoxynivalenol

The chromatographic system consisted of a Waters 2695 high-performance liquid chromatograph (Waters, Milford, CT, USA) with a Waters 2996 Photodiode Array Detector with a Nova Pak C-18 column (300 mm × 3.9 mm) for DON (λ_max_ = 224 nm) analysis. Quantification of the toxin was performed by measuring the peak areas at the retention time according to the relevant calibration curve. The limit of detection was 0.001 µg/g for DON.

### 3.7. Statistical Analysis

Data on DON concentrations in various tissue samples were evaluated for statistically significant differences employing the Tukey-test. Values are presented as medians and ranges. All statistical analyses were conducted using the Statistica Package for Social Science program.

## 4. Conclusions

After 7-day exposure to DON (term I) among tissue samples the liver was the only organ where DON could be detected. Median DON concentrations decreased in the order from the ileum > duodenum > jejunum for the small intestine specimens and the cecum > ascending colon > liver > descending colon > transverse colon for the large intestine and liver samples. Maximum carry-over rates reached the following levels: 0.037 (term VI) for the ileum (the small intestine), 0.049 (term V) for the ascending colon and 0.046 (term VI) for the cecum (the large intestine) and 0.081 (term I) for the liver. In conclusion, the results show how dose and exposure time may affect the distribution of DON in the gastrointestinal tract of gilts after *per os* toxin application.

## References

[1] Gräfenhan T., Patrick S.K., Roscoe M., Trelka R., Gaba D., Chan J.M., McKendry T., Clear R.M., Tittlemier S.A. (2013). *Fusarium* damage in cereal grains from Western Canada. 1. Phylogenetic analysis of moniliformin-producing *Fusarium* species and their natural occurrence in mycotoxin-contaminated wheat, oats, and rye. J. Agric. Food Chem..

[2] Juan C., Ritieni A., Mañes J. (2013). Occurrence of *Fusarium* mycotoxins in Italian cereal and cereal products from organic farming. Food Chem..

[3] Slikova S., Gavurnikova S., Sudyova V., Gregova E. (2013). Occurrence of deoxynivalenol in wheat in Slovakia during 2010 and 2011. Toxins.

[4] Wiśniewska H., Stępień Ł., Waśkiewicz A., Beszterda M., Góral T., Belter J. (2013). Toxigenic *Fusarium* species infecting wheat heads in Poland. Cent. Eur. J. Biol..

[5] Goliński P., Waśkiewicz A., Wiśniewska H., Kiecana I., Mielniczuk E., Gromadzka K., Kostecki M., Bocianowski J., Rymaniak E. (2010). Reaction of winter wheat (*Triticum aestivum* L.) cultivars to infection with *Fusarium* spp.: Mycotoxin contamination in grain and chaff. Food Add. Contam..

[6] Waśkiewicz A., Gromadzka K., Wiśniewska H., Goliński P. (2008). Accumulation of zearalenone in genotypes of spring wheat after inoculation with *Fusarium culmorum*. Cereal Res. Commun..

[7] Waśkiewicz A., Morkunas I., Bednarski W., Mai V.-C., Formela M., Beszterda M., Wiśniewska H., Goliński P. (2014). Deoxynivalenol and oxidative stress indicators in winter wheat inoculated with *Fusarium graminearum*. Toxins.

[8] Grenier B., Bracarense A.-P.F.L., Schwartz H.E., Lucioli J., Cossalter A.-M., Moll W.-D., Schatzmayr G., Oswald I.P. (2013). Biotranformation approaches to alleviate the effects induced by *Fusarium* mycotoxins in swine. J. Agric. Food Chem..

[9] Gajęcka M., Rybarczyk L., Jakimiuk E., Zielonka Ł., Obremski K., Zwierzykowski W., Gajęcki M. (2012). The effect of experimental long-term exposure to low-dose zearalenone on uterine histology in sexually immature gilts. Exp. Toxicol. Pathol..

[10] Girish C.K., MacDonald E.J., Scheinin M., Smith T.K. (2008). Effects of feedborne *Fusarium* mycotoxins on brain regional neurochemistry of turkeys. Poult. Sci..

[11] Wegulo S.N. (2012). Factors influencing deoxynivalenol accumulation in small grain cereals. Toxins.

[12] SCOOP (2003). Collection of Occurrence Data of *Fusarium* Toxins in Food and Assessment of Dietary Intake by the Population of EU Member States. Reports on Tasks for Scientific Cooperation. http://ec.europa.eu/food/fs/scoop/task3210.pdf.

[13] Waśkiewicz A., Irzykowska L., Bocianowski J., Koralewski Z., Kostecki M., Weber Z., Goliński P. (2010). Occurrence of *Fusarium* fungi and mycotoxins in marketable asparagus spears. Polish J. Environ. Stud..

[14] Koch H.-J., Pringas C., Maerlaender B. (2006). Evaluation of environmental and management effects on *Fusarium* head blight infection and deoxynivalenol concentration in the grain of winter wheat. Eur. J. Agron..

[15] Sobrowa P., Adam V., Vasatkova A., Beklova M., Zeman L., Kizek R. (2010). Deoxynivalenol and its toxicity. Interdiscip. Toxicol..

[16] Nagy C.M., Fejer S.N., Berek L., Molnar J., Viskolcz B. (2005). Hydrogen bondings in deoxynivalenol (DON) conformations—A density functional study. J. Mol. Struct..

[17] Döll S., Schrichx J.A., Dänicke S., Fink-Gremmels J. (2009). Interactions of deoxynivalenol and lipopolysaccharides on cytokine excretion and mRNA expression in porcine hepatocytes and Kupffer cell enriched hepatocyte. Toxicol. Lett..

[18] Pestka J.J. (2007). Deoxynivalenol: Toxicity, mechanisms and animal health risks. Anim. Feed Sci. Technol..

[19] Pestka J.J. (2010). Deoxynivalenol: Mechanisms of action, human exposure, and toxicological relevance. Arch. Toxicol..

[20] Bonnet M.S., Roux J., Mounien L., Dallaporta M., Troadec J.-D. (2012). Advances in deoxynivalenol toxicity mechanisms: The brain as a target. Toxins.

[21] Pestka J.J., Smolinski A.T. (2005). Deoxynivalenol: Toxicology and potential effects on humans. J. Toxicol. Environ. Health B Crit. Rev..

[22] Awad W.A., Aschenbach J.R., Setyabudi F.M.C.S., Razzazi-Fazeli E., Böhm J., Zentek J. (2007). *In vitro* effects of deoxynivalenol on small intestinal D-glucose uptake and absorption of deoxynivalenol across the isolated jejunal epithelium of laying hens. Poult. Sci..

[23] Arnold D.L., McGuire P.F., Nera E.A, Karpinski K.F., Bickis M.G., Zawidzka Z.Z., Fernie S., Vesonder R.F. (1986). The toxicity of orally administered deoxynivalenol (vomitoxin) in rats and mice. Food Chem. Toxicol..

[24] Maresca M., Mahfoud R., Garmy N., Fantini F. (2002). The mycotoxin deoxynivalenol affects nutrient absorption in human intestinal epithelial Cells. J. Nutr..

[25] Pinton P., Tsybulskyy D., Lucioli J., Laffitte J., Callu P., Lyazhri F., Grosjean F., Bracarense A.P., Kolf-Clauw M., Oswald I.P. (2012). Toxicity of deoxynivalenol and its acetylated derivatives on the intestine: Differential effects on morphology, barrier function, tight function proteins, and mitogen-activated protein kinases. Toxicol. Sci..

[26] Bensassi F., El Golli-Bennour E., Abid-Essefi S., Bouaziz C., Hajlaoui M.R., Bacha H. (2009). Pathway of deoxynivalenol-induced apoptosis in human colon carcinoma cells. Toxicology.

[27] Diesing A.K., Nossol C., Dänicke S., Walk N., Post A., Kahlert S., Rothkötter H.J., Kluess J. (2011). Vulnerability of polarised intestinal porcine epithelial cells to mycotoxin deoxynivalenol depends on the route of application. PLoS ONE.

[28] Bouhet S., Oswald I.P. (2005). The effects of mycotoxins, fungal food contaminants, on the intestinal epithelial cell-derived innate immune response. Vet. Immunol. Immunopathol..

[29] Sergent T., Parys M., Garsou S., Pussemier L., Schneider Y.J., Larondelle Y. (2006). Deoxynivalenol transport across human intestinal Caco-2 cells and its effects on cellular metabolism at realistic intestinal concentrations. Toxicol. Lett..

[30] Seeling K., Dänicke S., Valenta H., van egmond H.P., Schothorst R.C., Jekel A.A., Lebzien P., Schollenberger M., Razzazi-Fazeli E., Flachowsky G. (2006). Effects of *Fusarium* toxin-contaminated wheat and feed intake level on the biotransformation and carry-over of deoxynivalenol in dairy cows. Food Addit. Contam..

[31] Gutzwiller A. (2010). Effects of deoxynivalenol (DON) in the lactation diet on the feed intake and fertility of sows. Mycotoxin Res..

[32] Collins T.F.X., Sprando R.L., Black T.N., Olejnik N., Eppley R.M., Hines F.A., Rorie J., Ruggles D.I. (2006). Effects of deoxynivalenol (DON, vomitoxin) on in utero development in rats. Food Chem. Toxicol..

[33] Prelusky D.B., Gerdes R.G., Underhill K.L., Rotter B.A., Jui P.Y., Trenholm H.L. (1994). Effects of low-level dietary deoxynivalenol on the hematological and clinical parameters of the pig. Nat. Toxins.

[34] Swamy H.V.L.N., Smith T.K., MacDonald E.J., Boermans H.J., Squires E.J. (2002). Effects of feeding a blend of grains naturally contaminated with *Fusarium* mycotoxins on growth and immunological measurements of starter pigs, and the efficacy of a polymeric glucomannan mycotoxin adsorbent. J. Anim. Sci..

[35] Bretz M., Beyer M., Cramer B., Knecht A., Humpf H.-U. (2006). Thermal degradation of the *Fusarium* mycotoxin deoxynivalenol. J. Agric. Food Chem..

[36] Dänicke S., Valenta H., Döll S. (2004). On the toxicokinetics and the metabolism of deoxynivalenol (DON) in the pig. Arch. Anim. Nutr..

[37] Zielonka Ł., Wiśniewska M., Gajecka M., Obremski K., Gajecki M. (2009). Influence of low doses of deoxynivalenol on histopathology of selected organs of pigs. Pol. J. Vet. Sci..

[38] Goyarts T., Dänicke S., Valenta H., Ueberschär K.H. (2007). Carry-over of *Fusarium* toxins (deoxynivalenol and zearalenone) from naturally contaminated wheat to pigs. Food Addit. Contam..

[39] Dänicke S., Brezina U. (2013). Kinetics and metabolism of the Fusarium toxin deoxynivalenol in farm animals: Consequences for diagnosis of exposure and intoxication and carry over. Food Chem. Toxicol..

[40] Eriksen G.S., Pettersson H., Lindberg J.E. (2003). Absorption, metabolism and excretion of 3-acetyl DON in pigs. Arch. Tierernähr..

[41] Gajęcka M., Stopa E., Tarasiuk M., Zielonka Ł., Gajęcki M. (2013). The expression of type-1 and type-2 nitric oxide synthase in selected tissues of the gastrointestinal tract during mixed mycotoxicosis. Toxins.

[42] Silva-Campa E., Mata-Haro V., Mateu E., Hernández J. (2012). Porcine reproductive and respiratory syndrome virus induces CD4^+^CD8^+^CD25^+^Foxp3^+^ regulatory T cells (Tregs). Virology.

[43] Döll S., Dänicke S., Valenta H. (2008). Residues of deoxynivalenol (DON) in pig tissue after feeding mash or pellet diets containing low concentrations. Mol. Nutr. Food Res..

[44] Dänicke S., Goyarts T., Döll S., Grove N., Spolders M., Flachowsky G. (2006). Effects of the *Fusarium* toxin deoxynivalenol on tissue protein synthesis in pigs. Toxicol. Lett..

[45] Gupta A., Sharma A.C. (2004). Despite minimal hemodynamic alterations endotoxemia modulates NOS and p38-MAPK phosphorylation via metalloendopeptidases. Mol. Cell. Biochem..

[46] Grześk E., Grześk G., Koziński M., Stolarek W., Zieliński M., Kubica J. (2011). Nitric oxide as a cause and a potential place therapeutic intervention in hypo responsiveness vascular in early sepsis. Folia Cardiol..

[47] Castro M., Muńoz J.M., Arruebo M.P., Murillo M.D., Arnal C., Bonafonte J.I., Plaza M.A. (2012). Involvement of neuronal nitric oxide synthase (nNOS) in the regulation of migrating motor complex (MMC) in sheep. Vet. J..

[48] Lucioli J., Pinton P., Callu P., Laffitte J., Grosjean F., Kolf-Clauw M., Oswald I.P., Bracarense A.P.F.R.L. (2013). The food contaminant deoxynivalenol activates the mitogen activated protein kinases in the intestine: Interest of *ex vivo* models as an alternative to *in vivo* experiments. Toxicon.

[49] Alonso-Pozos I., Rosales-Torres A.M., Avalos-Rodriguez A., Vergara-Onofre M., Rosado-Garcia A. (2003). Mechanism of granulosa cell death during follicular atresia depends on follicular size. Theriogenology.

[50] Waché Y.J., Valat C., Postollec G., Bougeard S., Burel C., Oswald I.P., Fravalo P. (2009). Impact of deoxynivalenol on the intestinal microflora of pigs. Int. J. Mol. Sci..

[51] Alassane-Kpembi I., Kolf-Clauw M., Gauthier T., Abrami R., Abiola F.A., Oswald I.P., Puel O. (2013). New insights into mycotoxin mixtures: The toxicity of low doses of type B trichothecenes on intestinal epithelial cells is synergistic. Toxicol. Appl. Pharmacol..

[52] Awad W.A., Aschenbach J.R., Zentek J. (2012). Cytotoxicity and metabolic stress induced by deoxynivalenol in the porcine intestinal IPEC-J2 cell line. J. Anim. Physiol. Anim. Nutr..

[53] Grenier B., Loureiro-Bracarense A.-P., Lucioli J., Pacheco G.D., Cossalter A.-M., Moll W.-D., Schatzmayr G., Oswald I.P. (2011). Individual and combined effects of subclinical doses of deoxynivalenol and fumonisins in piglets. Mol. Nutr. Food Res..

[54] Weaver A.C., See M.T., Hansen J.A., Kim Y.B., de Souza A.L.P., Middleton T.F., Kim S.W. (2013). The use of feed additives to reduce the effects of aflatoxin and deoxynivalenol on pig growth, organ health and immune status during chronic exposure. Toxins.

[55] Awad W.A., Razzazi-Fazeli E., Böhm J., Ghareeb K., Zentek J. (2006). Effect of addition of a probiotic microorganism to broiler diets contaminated with deoxynivalenol on performance and histological alterations of intestinal villi of broiler chickens. Poult. Sci..

[56] Arunachalam C., Doohan F.M. (2013). Trichothecene toxicity in eukaryotes: Cellular and molecular mechanisms in plants and animals. Toxicol. Lett..

[57] Coppock R.W., Swanson S.P., Gelberg H.B., Koritz G.D., Hoffman W.E., Buck W.B. (1985). Preliminary study of the pharmacokinetics and toxicopathy of deoxynivalenol (vomitoxin) in swine. Am. J. Vet. Res..

[58] Kolf-Clauw M., Castellote J., Joly B., Bourges-Abella N., Raymond-Letron I., Pinton P. (2009). Development of a pig jejunal explant culture for studying the gastrointestinal toxicity of the mycotoxin deoxynivalenol: Histopathological analysis. Toxicol. in Vitro.

[59] D’Mello J.P.F., D’Mello J.P.F. (2000). Antinutritional factors and mycotoxins. Farm Animal Metabolism and Nutrition.

[60] Bracarense A.P., Lucioli J., Grenier B., Drociunas Pacheco G., Moll W.D., Schatzmayr G. (2012). Chronic ingestion of deoxynivalenol and fumonisin, alone or in interaction, induces morphological and immunological changes in the intestine of piglets. Br. J. Nutr..

[61] Pinton P., Nougayrede J.P., Del Rio J.C., Moreno C., Marin D.E., Ferrier L. (2009). The food contaminant deoxynivalenol, decreases intestinal barrier permeability and reduces claudin expression. Toxicol. Appl. Pharmacol..

[62] Vandenbroucke V., Croubels S., Martel A., Verbrugghe E., Goossens J., van Deun K. (2011). The mycotoxin deoxynivalenol potentiates intestinal inflammation by *Salmonella typhimurium* in porcine ileal loops. PLoS ONE.

[63] Davila A.-M., Blachier F., Gotteland M., Andriamihaja M., Benetti P.-H., Sanz Y., Tomé D. (2013). Re-print of “Intestinal luminal nitrogen metabolism: Role of the gut microbiota and consequences for the host”. Pharmacol. Res..

